# Development of Novel KASP Markers for Improved Germination in Deep-Sown Direct Seeded Rice

**DOI:** 10.1186/s12284-024-00711-1

**Published:** 2024-05-10

**Authors:** Nitika Sandhu, Jasneet Singh, Ade Pooja Ankush, Gaurav Augustine, Om Prakash Raigar, Vikas Kumar Verma, Gomsie Pruthi, Arvind Kumar

**Affiliations:** 1 Punjab Agricultural University, Ludhiana, Punjab 141004 India; 2 Delta Agrigenetics, Plot No. 99 & 100 Green Park Avenue, Village, Jeedimetla, Secunderabad, Telangana 500055 India

**Keywords:** Direct seeded rice, Early seedling vigor, Kompetitive Allele-Specific PCR, Mesocotyl length, Trait-linked marker

## Abstract

**Background:**

The lack of stable-high yielding and direct-seeded adapted varieties with better germination ability from deeper soil depth and availability of molecular markers are major limitation in achieving the maximum yield potential of rice under water and resource limited conditions. Development of high-throughput and trait-linked markers are of great interest in genomics-assisted breeding. The aim of present study was to develop and validate novel KASP (Kompetitive Allele-Specific PCR) markers associated with traits improving germination and seedling vigor of deep sown direct seeded rice (DSR).

**Results:**

Out of 58 designed KASP assays, four KASP assays did not show any polymorphism in any of the eleven genetic backgrounds considered in the present study. The 54 polymorphic KASP assays were then validated for their robustness and reliability on the F_1_s plants developed from eight different crosses considered in the present study. The third next validation was carried out on 256 F_3_:F_4_ and 713 BC_3_F_2:3_ progenies. Finally, the reliability of the KASP assays was accessed on a set of random 50 samples from F_3_:F_4_ and 80–100 samples from BC_3_F_2:3_ progenies using the 10 random markers. From the 54 polymorphic KASP, based on the false positive rate, false negative rate, KASP utility in different genetic backgrounds and significant differences in the phenotypic values of the positive (desirable) and negative (undesirable) traits, a total of 12 KASP assays have been selected. These 12 KASP include 5 KASP on chromosome 3, 1 on chromosome 4, 3 on chromosome 7 and 3 on chromosome 8. The two SNPs lying in the exon regions of *LOC_Os04g34290* and *LOC_Os08g32100* led to non-synonymous mutations indicating a possible deleterious effect of the SNP variants on the protein structure.

**Conclusion:**

The present research work will provide trait-linked KASP assays, improved breeding material possessing favourable alleles and breeding material in form of expected pre-direct-seeded adapted rice varieties. The marker can be utilized in introgression program during pyramiding of valuable QTLs/genes providing adaptation to rice under DSR. The functional studies of the genes *LOC_Os04g34290* and *LOC_Os08g32100* possessing two validated SNPs may provide valuable information about these genes.

**Supplementary Information:**

The online version contains supplementary material available at 10.1186/s12284-024-00711-1.

## Background

Rice (*Oryza sativa*) is a world’s major food crop. Considering the shortage of water and labor, and the advances in the agricultural mechanization, the direct seeded rice (DSR) appears as an alternative method of rice cultivation. The poor seed germination, serious weed infestation, and low seedling vigor are one among the major problems leading substantial yield loss in DSR cultivation system. The success of direct seeding relies strongly on the development of rice varieties with robust crop establishment (Kumar and Ladha 2011; Mahender et al. 2015). The broadcasting or surface seeding of rice may lead to poor establishment and the uneven crop stand due to predation, drought, rain splashing, greater vapour pressure gradient, and high temperature (Kumar and Ladha 2011; Yamauchi and Winn 1996). Instead, the deep sowing is an effective alternative method ensuring the seeds are fully protected, less vulnerable to pests and can access the available moisture from greater soil depths. The poor seedling emergence and establishment, low dry matter accumulation (Loeppky et al. 1989) caused by the deep sowing of rice greatly restrict the deep sown DSR technology. The mesocotyl length, along with seedling emergence and establishment are three important traits for determining high rice yields in deep sown DSR systems (Lee et al. 2017; Turner et al. 1982; Wu et al. 2015; Lu et al. 2016). The mesocotyl elongation is affected by various factors including light, water, soil depth and temperature. The plant hormones such as brassinosteroid (BR), abscisic acid (ABA), cytokinin (CTK), strigolactones (SLs), ethylene (ETH), gibberellin (GA), indole-3-acetic acid (IAA), and jasmonic acid (JA) play an important role in regulating the mesocotyl elongation. Earlier reports (Mahender et al. 2015; Turner et al. 1982; Dilday et al. 1990) suggested that the emergence rate of drill-seeded semi-dwarf rice genotypes is much lower and less uniform than the non-dwarf types genotypes with long mesocotyl. For the successful crop establishment under deep sown DSR, the DSR adapted rice varieties should have higher germination, faster seedling emergence with more vigorous growth and longer mesocotyl.

Quantitative Trait Loci (QTL) mapping facilitating the identification of targeted genomic regions associated with the favorable traits (Collard and Mackill 2008), gene pyramiding involving the simultaneous incorporation of multiple genes governing various traits into a single plant, resulting in varieties with a comprehensive array of desirable characteristics (Xu et al. 2017). The marker development enabling breeders to make accurate selections during the breeding process has emerged as a powerful strategy (Collard and Mackill 2008).

The advances in crop genome sequencing over few decades have had huge impacts on our knowledge to develop novel SNP (single nucleotide polymorphism) based molecular markers (Przewieslik-Allen et al. 2019) which have largely replaced the SSRs (simple sequence repeats) in cereal crop species (Semagn et al. 2014). Development of novel markers enables breeders to precisely identify and select the breeding lines/germplasm possessing desired traits. The efficient high-throughput ideal DNA markers possess the essential traits such as co-dominant inheritance, high genomic abundance and polymorphism, lower error rate, dense distribution, and seamless automation. The knock out effect of the widespread adoption of novel molecular markers have been seen in developing genomics-assisted breeding lines.

The SNP’s based markers have emerged as a powerful tool in various genetic applications including germplasm characterization and quality assessment, linkage mapping, association mapping, allele mining, marker-assisted selection and backcrossing, and genomic selection, (Rafalski 2002; Schlotterer 2004; Semagn et al. 2014). The high-throughput SNP genotyping platform, Kompetitive Allele-Specific PCR (KASP) assay has evolved as a global benchmark technology. KASP markers are being widely used for the genetic mapping and trait-specific marker development due to their low cost and low genotyping error rates, high reliability, and reproducibility (He et al. 2014; Ertiro et al. 2015; Rasheed et al. 2016; Tan et al. 2017).

A novel core-set of 110 KASP markers associated with traits improving grain yield and adaptability under DSR cultivation conditions was developed and validated (Sandhu et al. 2022). The developed KASP markers are now being routinely used in the genomics-assisted breeding programs for characterizing the breeding material with respect to important QTL/genes affecting grain yield, adaptability, biotic/abiotic stress tolerance/resistance under DSR cultivation conditions. A total of 71,311 KASP SNP markers with average density of 34 KASP/Mb from the RNA-Seq data have been developed for map-based cloning and the marker-assisted selection in maize (Chen et al. 2021). High density SNP arrays are available for the crop species including rice (Yu et al. 2014; Thomson et al. 2014; Chen et al. 2014; Singh et al. 2015), wheat (Allen et al. 2017), barley (Bayer et al. 2017), potato (Vos et al. 2015) and apple (Bianco et al. 2014, 2016). Considering the importance of KASP markers in genomics-assisted breeding, the objective of the present research was to develop and validate the SNP/allele specific trait-linked markers that target the genomic regions associated with improved germination and seedling vigour under deep-sown direct seeded rice cultivation conditions. To best of our knowledge this is the first study targeting development of trait-based SNP panel for the traits improving seedling vigor of rice under DSR. A set of core SNPs will be built via targeting variations in the already identified genomic region associated with DSR traits.

## Materials and Methods

The present study was carried out at School of Agricultural biotechnology, Punjab Agricultural University, Ludhiana, Punjab, India. To understand the genetic control of rice seedling vigour under DSR, genome wide association studies for multiple seedling traits in 684 accessions from the 3000 Rice Genomes (3 K-RG) population in both the laboratory and in the field at three planting depths (4, 8 and 10 cm) was carried out (Menard et al. 2021). Best donors with favourable allele and significant marker-trait associations (MTAs)/QTLs for mesocotyl length, percentage seedling emergence and shoot biomass in this panel were identified (Menard et al. 2021).

The seeds of donors including Aus344, N22, Kula Karuppan, NCS237, and IRGC 128442 were procured from IRRI (International Rice Research Institute) with the intention of using them as potential donors in a genomics-assisted breeding program. To assess polymorphism and to develop backcross and recombinant inbred populations for marker validation, five recipients' genetic backgrounds (PR126, PR121, PR128, PR129, PB1509) were carefully selected and used in the present study. The donors, recipient parents, F_1_s and the seven F_3_:F_4_ mapping populations viz. PR121/N22, PR126/N22, PR128/N22, PB1509/N22, PR121/Aus344, PR129/Aus344, and PR128/IRGC128442, and eight backcross mapping BC_3_F_2:3_ population N22/4*PR121, N22/4*PR126, N22/4*PR128, N22/4*PB1509, Aus344/4*PR121, Aus344/4*PR129, IRGC128442/4*PR126, and IRGC128442/4*PR128 were utilized to validate the KASP assay. The detailed information on the number of plants from each cross used to validate the KASP assay is presented in Additional file 1: Table S1.

### Phenotypic Evaluation of Parental Genotypes and Breeding Material

The donors including Aus344, N22, Kula Karuppan, NCS237, and IRGC 128442, the five recipients' including PR126, PR121, PR128, PR129, PB1509 and a set of 256 F_3_:F_4_ plants and 713 BC_3_F_2:3_ plants from the above-mentioned mapping populations were screened for the emergence from deeper soil depth in Kharif 2022 and 2023. The experimental design was completely randomized design (CRD) with three replications. Six seeds from each were sown in the plastic trays filled with soil at 4 cm, and 10 cm depth. In each tray, the donor possessing the longer mesocotyl length was kept as a positive check and PR126, PB 1509 was kept as a negative check. The seed sown at 4 cm depth was considered as a control for soil depth at 10 cm. The data on percent germination at a different soil depth, days to germination, mesocotyl length, root and shoot length were recorded. The data on percent germination at each sowing depth was recorded as (total number of seeds emerged out of the soil/total number of seeds planted) *100, days to germination was recorded in days, mesocotyl length with vernier calliper in mm, root and shoot length with centimetre scale.

### Statistical Analysis

The means were calculated from the replicated observations. Means were used to draw the frequency curves to know the phenotypic distribution of the traits. The data was pooled from both the years. The analysis of variance (ANOVA) for completely randomized design (CRD) was calculated in STAR (Statistical Tool for Agricultural Research) version 2.0.1. The ANOVA model for CRD was as follows:

$${\rm{Yij }} = {\upmu } + {\rm{ \alpha i }} + {\rm{ eij}}$$

where, Yij = Performance of the jth genotype in the ith block, μ = General mean, αi = Effect of ith treatment, eij = Error effect.

### Genotyping

#### Whole Genome Resequencing

The genomic DNA of the five donors (Aus344, N22, Kula Karuppan, NCS237, and IRGC 128442) and five recipient backgrounds (PR126, PR121, PR128, PR129, PB1509) were isolated using the modified CTAB method. The quality was examined using the gel electrophoresis. The high throughput whole genome resequencing was carried out at NGB diagnostic, New Delhi using Illumina HiSEQ 4000. The sequencing involved genomic DNA (gDNA) library preparation following the Illumina Truseq protocol v3, resulted in 150 bp paired-end short reads in fastq format. The initial output yielded a total of 4 Gb of raw sequence data. To refine this data, the following steps were employed in the processing pipeline. The entire procedure used for creating the core trait-linked KASP marker panel, integral for future genomics-assisted breeding initiatives includes sequencing, read processing, read alignments, variant calling and designing of KASP markers.

Sequencing, read processing, alignments, and variant calling: Utilizing the Illumina HiSeq 4000 platform, the paired-end sequencing was executed at NGB Diagnostics Private Limited, New Delhi, India. Following this, the read processing commenced. For the subsequent bioinformatics analysis, the initial step encompassed the quality check and elimination of Illumina adaptor sequences. Additionally, quality trimming was implemented on the reads, entailing the removal of adaptor-clipped reads containing Ns. Moreover, to achieve a minimum average Phred quality score of 20 across a ten-base window, 3′-end trimming was performed. Any reads concluding with a length below 20 bases were subsequently excluded from further analysis.

The *O. sativa* (version 7.0) reference sequence sourced from RGAP (Rice Genome Annotation Project, http://rice.plantbiology.msu.edu/pub/data/EukaryoticProjects/osativa/annotationdbs/pseudomolecules/version_7.0/all.dir/) was utilized for mapping. The sequencing reads were mapped against the reference genome using bwa tool (version 0.7.17-r1188). The default settings were used for the alignment parameters. The following analyses entirely incorporated the read pairs where only both the reads aligned as anticipated.

The Sam alignment format mapping files were converted into bam binary format using SAMtools (*version 0.1.19*) (Li et al. 2009). The Picard software (*version 1.48*) was used to detect and mark the duplicate entries in sorted bam files. The generated bam file served as an input file for the final variant calling using the Unified Genotyper software in GATK pipeline (Genome Analysis Toolkit, version 3.6). To facilitate comparative analysis and the identification of unique SNPs within donor parent variant files across all samples, the Bcftools tool (version 1.9) was applied to merge the variant files. Samples with a minor allele frequency (MAF) exceeding 2% and retaining at least 80% of the data were retained. The final step of variant calling involved filtering, accomplished using Vcftools (*version 0.1.17*) (Danecek et al. 2011).

#### Designing of KASP Markers

The KASP markers were designed using the offline Polymarker software (Ramirez-Gonzalez et al. 2015), while a combination of MAFFT, Exonerate, Primer3, Samtools, Bamtools, Bio-samtools, Blast software, and Glib 2.0 were employed within the system's path. The establishment of a reference genome database was accomplished through the BLAST tool, with subsequent indexing of the reference genome facilitated by samtools to generate a dedicated index file for the genome.

In past ten years efforts have been made at IRRI, Philippines and PAU, Ludhiana in the identification of donors and genomic regions associated with traits improving seedling vigor of rice under direct-seeded cultivation conditions (Menard et al. 2021; Sandhu et al. 2023). The genomic region spanning 16.67–24.65 Mb (*qSD*_*3.1*_) and 32.31–35.46 Mb (*qSD*_*3.2*_) on chromosome 3, 15.91–21.50 Mb on chromosome 4 (*qSD*_*4.1*_), 10.18–15.01 Mb (*qSD*_*7.1*_) and 20.93–24.26 Mb (*qSD*_*7.2*_) on chromosome 7, and 19.86–20.01 Mb (*qSD*_*8.1*_) on chromosome 8 showed association with % germination and mesocotyl length (Menard et al. 2021). Earlier Redoña and Mackill (1996) reported genomic regions from 16.84 to 29.58 Mb and 23.85–31.30 Mb on chromosome 3 that showed association with mesocotyl and coleoptile length in rice.

Variant calls pertaining to specific gene/QTL regions were extracted from the VCF files resulted from the SNP calling process. The flanking regions surrounded the SNPs were extracted from the reference genome using bedtools. These extracted SNP regions were then assembled into the desired format for the Polymarker software, incorporating essential details such as ID, chromosome number, and variant calls, along with 100-bp flanking regions on each side, formatted in CSV (Comma-separated values). These meticulously prepared files were subsequently employed as input data for the Polymarker software.

#### Filtering and Selection of KASP Markers

The markers located in the earlier identified genomic region associated with the traits improving seedling vigor of rice under DSR were screened. All the gene files for the reference genome were retrieved from RGAP. The selected markers then screened for the donor specificity using the merged variant file created using BCF tools. All the shortlisted markers were aligned with the reference genome using BLAST and the markers showing alignment at the multiple loci were rejected. Only the high specificity markers aligning at desired locus with low e-value were selected.

## Results

### Phenotyping

**Fig. 1:**
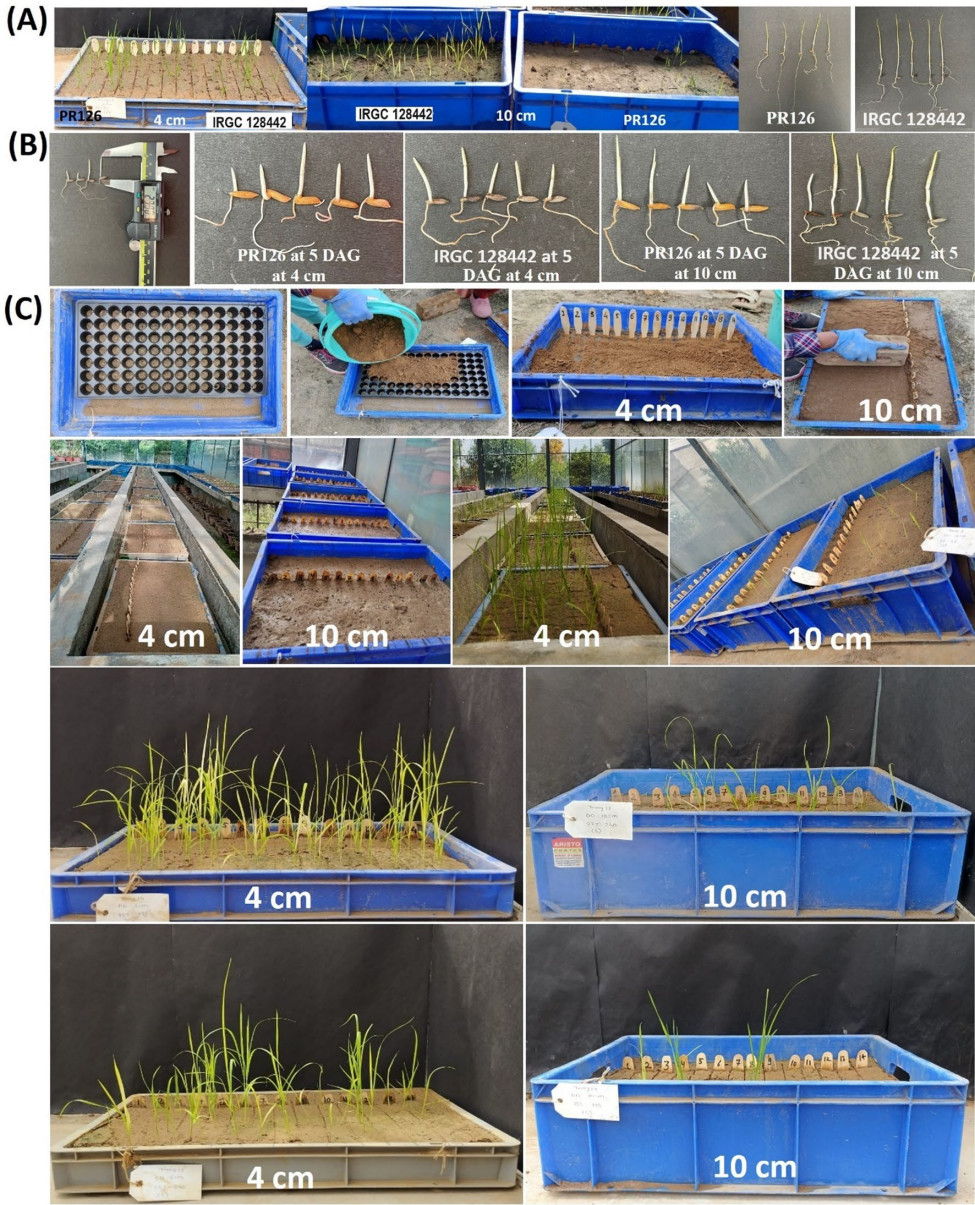
Phenotypic evaluation of F_3_:F_4_ and BC_3_F_2:3_ for different traits improving seedling vigor of rice under control (4 cm) and deep sown (10 cm) direct seeded cultivation conditions. **A** Phenotypic evaluation of parental lines (PR126; one of the recipient parent and IRGC 128442; one of the donor parent) at 4 cm and 10 cm of sowing depth. **B** Phenotypic variations of mesocotyl length in the PR26 and IRGC 128442 parental lines at 5 DAG (days after germination). **C** Phenotypic evaluation of F_3_:F_4_ and BC_3_F_2:3_ progenies under control (4 cm) and deep sown (10 cm) direct-seeded cultivation conditions under screen house conditions

The donors including Aus344, N22, Kula Karuppan, NCS237, and IRGC128442, the five recipients' including PR126, PR121, PR128, PR129, PB1509 and a set of 256 F_3_:F_4_ plants from the eight mapping populations viz. PR121/N22, PR126/N22, PR128/N22, PB1509/N22, PR121/Aus344, PR129/Aus344, and PR128/IRGC128442 and 713 BC_3_F_2:3_ plants from the eight backcross mapping population N22/4*PR121, N22/4*PR126, N22/4*PR128, N22/4*PB1509, Aus344/4*PR121, Aus344/4*PR129, IRGC128442/4*PR126, and IRGC128442/4*PR128 (Additional file 1: Table S1) were screened for different traits associated with emergence from deeper soil depth at 4 cm and 10 cm (Fig. 1). Significant phenotypic variations were observed for the traits measured in the present study in F_3_:F_4_ progenies (Table 1) and BC_3_F_2:3_ progenies (Table 2). The % germination of the F_3_:F_4_ plants from the eight mapping populations ranged from 8.3 to 91.67% at 4 cm and 0 to 83.33% at 10 cm of soil depth (Table 1). The % germination of the BC_3_F_2:3_ plants from the eight backcross populations ranged from 8.3 to 100% at 4 cm and 0 to 91.67% at 10 cm of soil depth (Table 2). The mesocotyl length varied from 1.4 to 2.0 cm at 4 cm and 2.8 to 5.0 cm at 10 cm soil depth in F_3_:F_4_ mapping populations (Table 1) and from 2.3 to 3.1 cm at 4 cm and 3.6 to 6.0 cm at 10 cm soil depth in backcross populations (Table 2). The root and shoot length of the F_3_:F_4_ plants ranged from 1.0 to 6.0 cm and 14.0 to 24.5 cm at 4 cm, respectively and from 2.0 to 7.8 and 14.0 to 26.7 cm at 10 cm of soil depth, respectively.

**Table 1:** Detailed description of analysis of variance, minimum, maximum, mean and coefficient of variations in 256 F_3_:F_4_ progenies

	Min	Max	Mean	StdDev	Pr (> Chisq)	Pr (< W)	Pr (> F)	CV (%)
%Germination_4 cm	0	91.67	43.91	22.52	0.0000	0.0000	0.0005	3.66
ML_4cm	0	6.5	5.58	1.36	0.0000	0.0000	0.0001	3.34
RL_4cm	0	11	4.67	1.98	0.0000	0.0000	0.0000	17.3
TL_4 cm	0	33	22.12	6.68	0.0000	0.0000	0.005	13
%Germination_10 cm	0	83.33	29	19.95	0.0000	0.0000	0.0003	5.54
ML_10 cm	0	10	7.42	3.16	0.0000	0.0000	0.0000	6.84
RL_10 cm	0	12	3.29	1.87	0.0000	0.0000	0.0000	10.39
TL_10 cm	0	35	21.31	9.52	0.0000	0.0000	0.0000	6.64

ML: mesocotyl length (cm), RL: root length (cm), TL: total plant length (cm), stdDev: standard deviation,W: wald test, F: F-test, CV: coefficient of variations (%)

**Table 2:** Detailed description of analysis of variance, minimum, maximum, mean and coefficient of variations in 713 BC_3_F_2:3_ progenies

	Min	Max	Mean	StdDev	Pr (> Chisq)	Pr (< W)	Pr (> F)	CV (%)
%Germination_4 cm	8.33	100	45.5	21.62	0.0000	0.0000	0.0003	3.21
ML_4cm	5.3	6.2	5.84	0.2096	0.0000	0.0000	0.0001	3.38
RL_4cm	2	10	4.48	1.53	0.0000	0.0000	0.0000	11.04
TL_4 cm	15	33	22.54	4.31	0.0000	0.0000	0.005	15.57
%Germination_10 cm	0	91.67	23.62	19.87	0.0000	0.0000	0.0003	5.54
ML_10 cm	0	10	6.94	3.59	0.0000	0.0000	0.0000	1.34
RL_10 cm	0	11	3.3	2.23	0.0000	0.0000	0.0000	5.47
TL_10 cm	0	34	19.28	10.5	0.0000	0.0000	0.0000	2.75

ML: mesocotyl length (cm), RL: root length (cm), TL: total plant length (cm), stdDev: standard deviation, W: wald test, F: F-test, CV: coefficient of variations (%)

### Genome Wide Discovery of Polymorphism Among Different Donors and Recipients

**Fig. 2:**
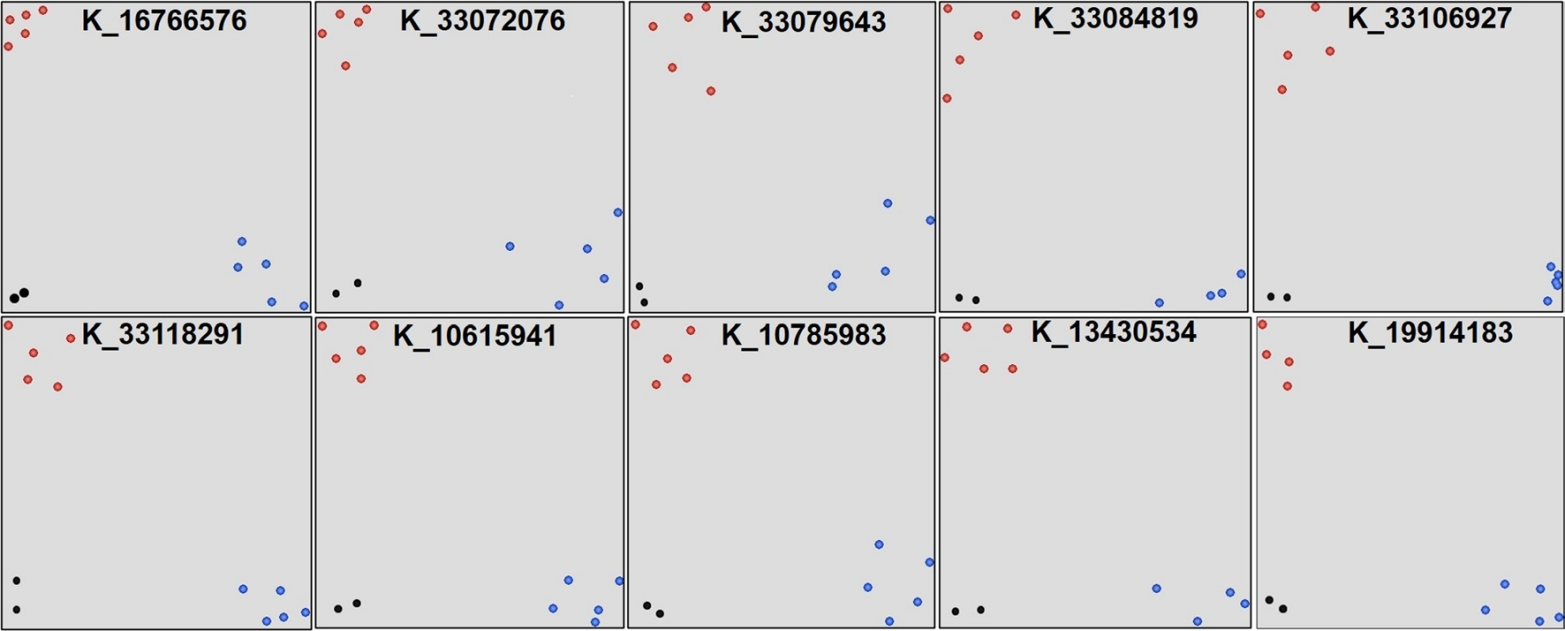
The pictorial representation of the KASP assays conducted on the 10 genotypes including 5 donors (Aus344, N22, Kula Karuppan, NCS237, IRGC 128442) and 5 recipient background (PR121, PR126, PR128, PR129, Pusa Basmati 1509) used to develop the breeding panel. Blue color indicates the donor allele, red color indicates the recipient allele and green color indicates the heterozygotes

The whole genome resequencing of eleven diverse genotypes including 5 donors (Aus344, N22, Kula Karuppan, NCS237, IRGC 128442) and 6 recipient background (PR121, PR126, PR128, PR129, MTU1010, Pusa Basmati 1509) resulted in a total of 41,00,81,779 paired end reads of 161 bp (Additional file 1: Table S2). In the eleven genotypes, the read based %GC content estimate ranged from 43 to 48% (Additional file 1: Table S2). The 98% of the filtered reads were mapped on rice Nipponbare reference genome. The average genome coverage was 98.22% with highest in Kula Karuppan (98.46%) and lowest in NCS237 (97.5%) (Additional file 1: Table S2). From the high-quality sequences, a total of variants at 10 × were 2,89,38,981 with average variant per sample 2,630,816 (Additional file 1: Table S2). The number of variants at 10 × was highest in Pusa Basmati 1509 (34,01,748) and lowest in Kula Karuppan (184,335). The genome sequence of all the 5 donors was compared with each of the six recipient backgrounds for the identification of SNPs. The designed KASP assays were very informative for the rice germplasm constituting 5 donors and 6 recipient backgrounds. Out of 58 designed KASP assays, four KASP assays (K_10517640, K_21414536, K_21515581, and K_19899355) did not show any polymorphism for any of the recipient background used in the present study. The few examples of KASP assays on the 10 parental genotypes is presented in Fig. 2. Of the total 54 polymorphic KASP, 45 KASP were localized within the MSUv7 gene models (http://rice.plantbiology.msu.edu), and 9 KASP markers were located within the intergenic regions (Additional file 1: Table S3). The 9 KASP markers located in the intergenic region of chromosome 7 (Additional file 1: Table S3). The highest quality SNPs were detected for the KASPs, K_33070058 (A—> C 33070058), K_33072076 (A—> T 33072076), K_33079643 (A—> C 33079643), K_33106927 (T—> C 33106927), K_20760206 (G—> A 20760206), K_20853559 (A—> T 20853559) and K_10923664 (C—> T 10923664) in IRGSP1.0 (International Rice Genome Sequencing Project (Additional file 1: Table S3).

The average physical distance between the two polymorphic KASP markers on chromosome 3 was 505 kb or ~ 2.070 cM for *qSD*_*3.1*_ and 13.16 kb or ~ 0.0539 cM for *qSD*_*3.2*_ considering 1 cM equal to ~ 244 kb (Chen et al. 2002). The average physical distance between the two polymorphic KASP markers in genomic region associated with *qSD*_*4.1*_ on chromosome 4 was 35.17 kb or ~ 0.144 cM. Further, this average distance was 228.86 kb (~ 0.978 cM), 293.43 kb (~ 1.203 cM) and for 30.17 kb (~ 0.124 cM) *qSD*_*7.1*_, *qSD*_*7.2*_ and *qSD*_*8.1*_, respectively.

### Quality Parameters of KASP Markers

The quality of each of 54 polymorphic KASP markers for the traits associated with seedling vigor was assessed based on the parameters such as utility, False positive rate (FPR) and False negative rate’ (FNR) of the KASP markers (Table 1). The quality was assessed on the 256 F_3_:F_4_ plants from seven populations and 713 BC_3_F_2:3_ plants derived from the eight backcrossed populations. The utility of the KASP markers ranged from all the six recipient backgrounds to only one or two different recipient backgrounds. The allelic effects of all the polymorphic KASP on the phenotypes of the F_3_:F_4_ and BC_3_F_2:3_ derived populations are described for the %germination and mesocotyl length traits in Table 3. The FPR and FNR of the KASP assays in F_3_:F_4_ mapping populations ranged from 0.0114 to 0.1316 and 0.0 to 0.0952, respectively (Table 1). While, in the BC_3_F_2:3_ populations the FPR and FNR of the KASP assays ranged from 0.0643 to 0.25 and 0.0055 to 0.1181, respectively (Table 3). The utility of KASP assays varies from all five genetic backgrounds to one background only. A total of 13 KASP assays showed utility in five recipient backgrounds, 15 KASP assays in any of the four recipient backgrounds, 5 KASP assays in any of the three recipient backgrounds, 8 KASP assays in any of the two recipient backgrounds and 13 KASP assays in any of the one recipient backgrounds. Out of the 13 KASP that showed utility in all 5 genetic backgrounds, 8 KASP were present on chromosome 3, three were on chromosome 7 and two KASP on chromosome 8. Out of 15 KASP assays that were polymorphic for four genetic backgrounds three KASP belonged to chromosome 3, one KASP belonged to chromosome 4, seven KASP to chromosome 7 and four KASP to chromosome 8. Further, the 5 KASP that showed polymorphism with three recipient backgrounds were present on chromosome 3 (1 KASP), chromosome 4 (3 KASP) and chromosome 7 (1 KASP). The detailed information on each of the KASP markers showing utility to each of the six recipient backgrounds, their allelic interpretation FPR, FNR are presented in the Table 3.

**Table 3:** The quality control assessment results and the allelic effects of the 54 trait-linked KASP markers validated on the phenotypes of the 11 diverse genotypes, 256 F_3_:F_4_ and 713 BC_3_F_2:3_ progenies

Marker ID	Chr	Position	Ref allele	Positive allele	KASP Utility	NILs		RILs		NILs							RILs							
						KASP FPR	KASP FNR	KASP FPR	KASP FNR	Total number of genotypes tested	Frequency (%)		Phenotypic mean_% germination		Phenotypic mean_ML		Total number of genotypes tested	Frequency (%)		Phenotypic mean_% germination		Phenotypic mean_ML		Significance level
											Negative trait	Positive trait	Negative trait	Positive trait	Negative trait	Positive trait		Negative trait	Positive trait	Negative trait	Positive trait	Negative trait	Positive trait	
K_16766576	3	16,766,576	C	T	PR121, PR126, PR128, PR129, PB1509	0.2308	0.0181	0.1286	0.0078	374	156 (41.7%)	166 (44.4%)	27.3	64.4	0.8	4.83	256	69 (27.0%)	129 (50.4%)	21.4	65.1	1.1	4.8	**
K_16856978	3	16,856,978	C	T	PR121, PR126, PR128, PR129, PB1509	0.0785	0.0258	0.0909	0.0081	274	131 (35.0%)	121 (32.4%)	22.4	78.9	1.2	5.8	187	61 (32.6%)	83 (44.4%)	15.0	75.3	0.9	5.64	***
K_19041692	3	19,041,692	C	T	PR126, PR128, PR129, PB1509	0.0971	0.0055	0.0380	0.0090	264	125 (33.4%)	110 (29.4%)	24. 6	74.4	1.14	5.64	207	60 (29.0%)	92 (44.4%)	22.5	77.0	0.8	5.58	***
K_20267111	3	20,267,111	T	C	PR126, PB1509	0.2201	0.0192	0.0506	0.0700	118	48 (40.7%)	47 (39.8%)	26.7	64.5	0.96	4.8	187	50 (26.7%)	75 (40.1%)	27.3	68.4	1.2	4.5	**
K_20957428	3	20,957,428	A	T	PR126, PB1509	0.1807	0.0261	0.0260	0.0392	86	29 (33.7%)	45 (52.3%)	27.1	65.6	1.2	4.44	70	29 (41.4%)	26 (37.1%)	27.2	61.4	1.47	4.92	***
K_24626773	3	24,626,773	T	G	PR121, PR126, PR129, PB1509	0.1180	0.0278	0.0395	0.0792	86	32 (37.2%)	44 (51.2%)	29.2	67.1	1.4	4.0	70	23 (32.9%)	35 (50%)	22.6	67.5	1.1	4.25	**
K_24639959	3	24,639,959	C	T	PR121, PR126	0.1829	0.0196	0.0366	0.0784	118	48 (40.7%)	44 (37.3%)	24.0	57.5	0.57	3.85	44	20 (45.5%)	18 (40.9%)	21.8	56.4	1.3	3.89	*
K_33013006	3	33,013,006	A	C	PR121, PR126, PR129	0.1598	0.0270	0.0400	0.0583	86	40 (46.5%)	29 (33.7%)	15.8	55.4	0.8	3.24	113	33 (29.2%)	47 (41.6%)	23.8	65.3	0.88	3.65	*
K_33070058	3	33,070,058	A	C	PR121, PR126, PR128, PR129	0.1036	0.0221	0.0247	0.0900	219	103 (47.0%)	83 (37.9%)	18.7	81.1	0.84	5.5	140	44 (31.4%)	52 (37.1%)	18.6	77.7	1.2	5.2	**
K_33072076	3	33,072,076	A	T	PR121, PR126, PR128, PR129, PB1509	0.1097	0.0226	0.0233	0.0400	374	154 (41.2%)	132 (35.3%)	14.3	81.5	0.55	5.19	256	86 (33.6%)	100 (39.1%)	16.4	80.9	0.9	5.5	***
K_33079643	3	33,079,643	A	C	PR121, PR126, PR128, PR129, PB1509	0.1097	0.0226	0.1266	0.0952	374	185 (49.5%)	174 (46.5%)	19.7	83.3	0.8	5.2	256	79 (30.9%)	105 (41.0%)	19.7	73.9	1.2	5	***
K_33084819	3	33,084,819	G	A	PR121, PR126, PR128, PR129, PB1509	0.1722	0.0866	0.1013	0.0700	274	121 (44.2%)	114 (41.6%)	28.4	66.9	1.19	4.32	49	14 (28.6%)	23 (46.9%)	27.0	65.1	1.1	3.89	*
K_33106927	3	33,106,927	T	C	PR121, PR126, PR128, PR129, PB1509	0.1645	0.0714	0.1111	0.0600	374	152 (40.6%)	126 (33.7%)	28.8	55.9	1.2	4.51	256	81 (31.6%)	100 (39.1%)	28.5	64.4	1.08	4.2	**
K_33107252	3	33,107,252	T	G	PR121, PR126, PR128, PR129, PB1509	0.0798	0.0588	0.0127	0.0469	374	163 (43.6%)	119 (31.8%)	27.01	80.0	1.1	5.84	256	79 (30.9%)	127 (49.6%)	21.0	77.0	0.9	5.7	***
K_33117352	3	33,117,352	C	T	PR128, PR129	0.1316	0.0738	0.0732	0.0157	146	60 (41.1%)	52 (35.6%)	25.69	71.7	1.5	4.46	138	37 (26.8%)	79 (57.3%)	25.6	68.5	1.04	4.68	**
K_33118291	3	33,118,291	T	C	PR121, PR126, PR128, PR129, PB1509	0.1558	0.0543	0.0759	0.0504	374	154 (41.2%)	129 (34.2%)	22.4	69.6	1.2	4.46	256	79 (30.9%)	121 (47.3%)	24.0	65.1	1.6	4.8	**
K_20594561	4	20,594,561	A	G	PR128, PR129	0.1831	0.0635	0.0759	0.0496	146	50 (34.3%)	57 (39.0%)	16.4	65.5	0.88	3.19	138	38 (27.5%)	78 (56.5%)	30.2	67.8	1.1	4.42	**
K_20716230	4	20,716,230	T	C	PR128, PR129	0.1788	0.0602	0.0513	0.0667	146	55 (37.7%)	58 (39.7%)	16.5	64.7	1.04	4.55	138	34 (24.6%)	82 (59.4%)	26.1	60.6	1.4	3.82	*
K_20760206	4	20,760,206	G	A	PR121, PR126, PR129	0.2138	0.0606	0.0494	0.0325	131	69 (52.7%)	43 (32.8%)	18.4	61.6	0.66	4.42	118	32 (27.1%)	63 (53.4%)	28.2	66.2	1.26	4.74	**
K_20771274	4	20,771,274	G	A	PR121, PR126, PR128, PR129	0.1218	0.0552	0.0641	0.0583	86	35 (40.7%)	32 (37.2%)	29.3	74.1	1.11	5.01	49	14 (28.6%)	29 (59.2%)	25.4	78.9	1.1	5.2	***
K_20823126	4	20,823,126	G	A	PR129	0.1972	0.0687	0.0741	0.0413	45	24 (53.3%)	16 (35.6%)	24.2	66.0	0.65	4.27	42	11 (26.2%)	28 (66.7%)	27.1	61.0	1.38	4.54	**
K_20835306	4	20,835,306	T	A	PR129	0.1875	0.0465	0.0667	0.0750	45	26 (57.8%)	14 (31.1%)	22.0	73.8	0.66	3.84	42	10 (23.8%)	27 (64.3%)	19.2	70.9	1.2	4	*
K_20853559	4	20,853,559	A	T	PR128, PR129	0.1467	0.0476	0.0769	0.0325	100	28 (28%)	43 (43%)	29.5	64.8	1	4.1	96	26 (27.1%)	50 (52.1%)	32.2	61.1	1.5	3.74	*
K_20862639	4	20,862,639	C	A	PR121, PR126, PR129	0.1837	0.0687	0.0649	0.0583	163	89 (54.6%)	49 (30.1%)	15.5	54.7	1.3	3.3	113	32 (28.3%)	47 (41.5%)	25.5	65.6	1.28	3.7	*
K_20875927	4	20,875,927	A	G	PR121, PR126, PR128	0.1812	0.0620	0.0633	0.0328	188	79 (42.0%)	54 (28.7%)	19.6	61.7	0.5	3.6	118	32 (27.1%)	48 (40.7%)	29.3	60.5	1.5	3.89	*
K_10023203	7	10,023,203	G	T	PR121, PR128, PR129, PB1509	0.1644	0.0551	0.0909	0.0504	256	110 (43.0%)	94 (36.7%)	21.1	55.6	0.9	3.4	118	29 (24.6%)	65 (55.1%)	27.0	58.7	1.1	3.73	**
K_10068231	7	10,068,231	T	A	PR121, PR128, PR129, PB1509	0.1645	0.0775	0.0641	0.0410	256	106 (41.4%)	90 (35.2%)	19.8	58.8	1.2	4.1	118	31 (26.3%)	62 (52.5%)	28.2	58.5	1.6	3.94	**
K_10131516	7	10,131,516	T	C	PR126	0.1901	0.0448	0.0247	0.0583	370	156 (42.2%)	125 (33.8%)	22.2	75.4	1.1	5	71	25 (35.2%)	34 (47.9%)	23.7	69.3	1.4	4.8	**
K_10280980	7	10,280,980	T	A	PR126	0.1656	0.0794	0.0494	0.0598	370	144 (38.9%)	132 (35.7%)	21.6	68.8	0.9	4.2	71	22 (31.0%)	33 (46.5%)	26.2	66.4	1.4	4.56	**
K_10615941	7	10,615,941	G	T	PR121, PR126, PR128, PR129, PB1509	0.1447	0.0726	0.0732	0.0583	100	59 (59%)	27 (27%)	26.6	67.2	0.86	4.6	118	33 (28.0%)	63 (53.4%)	28.3	65.0	1.8	4.73	**
K_10785983	7	10,785,983	G	T	PR121, PR126, PR128, PR129, PB1509	0.1958	0.0385	0.0641	0.0417	713	312 (43.8%)	290 (40.7%)	22.3	64.4	0.6	4.8	256	78 (30.5%)	120 (46.9%)	21.1	64.9	1.1	3.89	*
K_10852628	7	10,852,628	A	T	PR126	0.1769	0.0543	0.0750	0.0410	370	158 (42.7%)	136 (36.8%)	18.9	60.4	0.87	4.16	22	7 (31.8%)	7 (31.8%)	21.4	57.1	1.1	3.93	*
K_10923664	7	10,923,664	C	T	PR126	0.1918	0.0534	0.0494	0.0417	370	166 (44.9%)	130 (35.1%)	19.9	76.8	1.1	4.29	22	11 (50.0%)	5 (22.7%)	30.3	69.7	1.4	4.45	**
K_11112584	7	11,112,584	C	T	PR126	0.1633	0.0635	0.0897	0.0413	402	195 (48.5%)	165 (41.0%)	22.8	72.7	1.4	5.1	22	8 (36.4%)	6 (27.3%)	20.8	69.7	1.2	4.9	**
K_11280410	7	11,280,410	C	A	PR126	0.1818	0.0303	0.0633	0.0420	402	186 (46.3%)	161 (40.1%)	21.1	70.5	1.1	4.7	22	10 (45. 5%)	5 (22.7%)	27.5	71.2	1.3	4.89	**
K_11362407	7	11,362,407	C	T	PR126	0.1757	0.0692	0.1154	0.0480	402	177 (44.0%)	170 (42.3%)	19.9	69.9	1.6	4	22	8 (36.4%)	8 (36.4%)	24.0	68.7	1.13	4.9	**
K_11964495	7	11,964,495	C	A	PR126	0.2168	0.0602	0.0759	0.0583	402	159 (39.6%)	170 (42.3%)	22.8	71.4	1.1	4.5	22	10 (45.5%)	5 (22.7%)	30.0	65.0	1.2	4.92	**
K_12452138	7	12,452,138	G	T	PR126	0.1600	0.0703	0.0235	0.0504	402	167 (41.5%)	154 (38.3%)	20.3	74.8	0.94	4.9	22	9 (40.9%)	8 (36.4%)	25.0	68.9	0.99	4.86	***
K_12689303	7	12,689,303	C	T	PR126	0.1918	0.0923	0.0341	0.0690	402	175 (43.5%)	181 (45.0%)	22.5	78.3	1.4	5.2	22	11 (50.0%)	4 (18.2%)	28.0	68.8	1.4	4.89	**
K_12922335	7	12,922,335	A	G	PR126, PB1509	0.2113	0.0775	0.0345	0.0769	457	197 (43.1%)	188 (41.1%)	29.9	71.6	1.2	4.89	69	31 (44.9%)	26 (37.7%)	27.9	71.2	1.5	4.91	**
K_13314239	7	13,314,239	T	C	PR121, PR126, PR128, PR129	0.1180	0.0123	0.0417	0.0156	658	313 (47.6%)	293 (44.5%)	26.1	81.6	1.5	5.4	209	59 (28.2%)	111 (53.1%)	23.0	75.9	1.3	5.2	***
K_13430534	7	13,430,534	G	A	PR121, PR126, PR128, PR129, PB1509	0.0719	0.0649	0.0704	0.0476	713	320 (44.9%)	305 (42.8%)	19.34	81.6	0.5	5.7	256	72 (28.1%)	128 (50%)	19.95	80.9	0.98	5.5	***
K_13565675	7	13,565,675	C	G	PR121, PR126, PB1509	0.0643	0.1000	0.0435	0.0397	567	240 (42.3%)	244 (43.0%)	21.7	79.7	1.2	5.8	118	36 (30.5%)	40 (33.9%)	20.8	78.9	1.1	5.45	***
K_13832487	7	13,832,487	A	G	PR121, PR126, PR128, PR129	0.1258	0.0183	0.0235	0.0000	658	291 (44.2%)	282 (42.9%)	25.9	76.7	1	5.48	219	63 (28.8%)	103 (47.0%)	20.8	77.9	0.99	5.1	**
K_14641954	7	14,641,954	G	A	PR121, PR126, PR128, PR129	0.1258	0.0183	0.0233	0.0198	658	286 (43.5%)	294 (44.7%)	21.1	78.9	1	5.1	219	67 (30.6%)	86 (39.3%)	23.1	78.7	1.1	5.3	***
K_14713452	7	14,713,452	G	A	PR121, PR126, PR128, PR129	0.1069	0.0120	0.0114	0.0094	658	281 (42.7%)	292 (44.4%)	19.5	81.4	0.7	5.5	219	67 (30.6%)	85 (38.8%)	21.3	81.3	0.94	5.44	***
K_14928973	7	14,928,973	C	T	PR121, PR126, PR128, PR129	0.1133	0.0121	0.0370	0.0171	658	288 (43.8%)	277 (42.1%)	24.4	77.5	1.2	4.9	219	62 (28.3%)	99 (45.2%)	21.8	77.4	1.4	5	**
K_22001478	7	22,001,478	G	A	PR126	0.2500	0.0823	0.0909	0.0336	402	180 (44.8%)	167 (41.5%)	21.6	67.9	1.5	4.2	22	9 (40.9%)	6 (27.3%)	21.8	68.2	1.6	4.7	**
K_19899233	8	19,899,233	C	T	PR121, PR126, PR128, PR129, PB1509	0.1020	0.0118	0.0441	0.0076	274	106 (38.7%)	114 (41.6%)	25.9	78.7	0.9	5.1	187	58 (31.0%)	91 (48.7%)	23.1	78.5	1.1	5	**
K_19900483	8	19,900,483	T	A	PR121, PR126, PR128, PR129	0.0682	0.1181	0.0435	0.0156	219	106 (48.4%)	92 (42.0%)	24.9	71.9	1.1	5.6	140	40 (28.6%)	70 (50%)	21.7	74.4	1.2	5.25	***
K_19903800	8	19,903,800	C	A	PR121, PR126, PR129, PB1509	0.2349	0.0701	0.0282	0.0236	173	75 (43.4%)	71 (41.0%)	30.6	68.2	0.9	4.8	133	41 (30.8%)	59 (44.4%)	22.4	66.2	1.17	4.73	**
K_19914183	8	19,914,183	T	C	PR121, PR126, PR129, PB1509	0.0893	0.0347	0.0267	0.0160	273	104 (38.1%)	96 (35.2%)	25.6	80.2	0.8	5.4	256	75 (29.3%)	125 (48.8%)	24.9	79.6	1.1	5.32	***
K_19914306	8	19,914,306	C	T	PR121, PR126, PR128, PR129, PB1509	0.0814	0.0827	0.0519	0.0159	274	120 (43.8%)	100 (36.5%)	30.7	78.2	1.1	5.1	187	62 (33.2%)	85 (45.5%)	25.7	79.4	1.34	5.1	***
K_19917333	8	19,917,333	G	A	PR121, PR126, PR129, PB1509	0.0710	0.0930	0.0667	0.0238	172	78 (45.4%)	61 (35.5%)	31.0	68.5	1.2	4.6	91	37 (40.7%)	31 (34.1%)	36.1	59.6	1.6	4.0	**

Chr: chromosome, bp: base pair, Ref allele: allele present in the reference genome, positive allele: allele present in the donor parent, FPR: false positive rates, FNR: false negative rates, frequency (%) negative trait: number (percent to the total) of the breeding lines possessing recipient parent allele, frequency (%) positive trait: number (percent to the total) of the breeding lines possessing donor parent allele, phenotypic mean negative trait: mean value of the breeding lines possessing recipient parent allele, phenotypic mean positive trait: mean value of the breeding lines possessing donor parent allele, KASP utility: the percentage of a prospective background across which the SNP marker could be used to introgress the positive allele associated with the trait of interest, False Positive Rate’ (FPR): the proportion of breeding lines with recipient allele but identified as not having an unfavorable/recipient allele of the SNP marker. It was calculated as the number of breeding lines withOUT recipient allele/Total number of breeding lines with recipient allele, False Negative Rate (FNR): the proportion of breeding lines with donor allele but identified as not having the desired QTL/donor allele. It was calculated as: # number of breeding lines with-OUT favorable allele/Total number of breeding lines with donor allele. The significance level indicates the allelic effects of the KASP assays on the mean phenotypic values of the NILs and RILs estimated using Kruskal–Wallis test. *Significance at < 5% level, **significance at < 1% level, ***significance at < 0.1% level

### Genetic Relationship

**Fig. 3:**
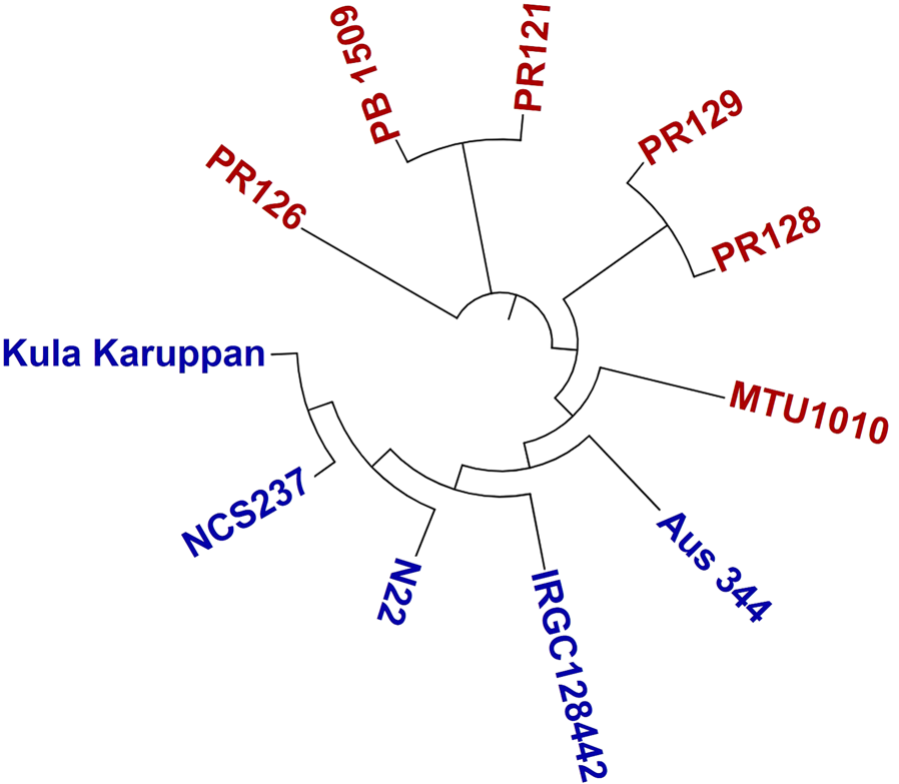
The genetic diversity analysis of the 11 genotypes including 5 donors (Aus344, N22, Kula Karuppan, NCS237, IRGC 128442) and 6 recipient background (PR121, PR126, PR128, PR129, MTU1010, Pusa Basmati 1509) using the whole genome resequencing data

The genetic relationship among the eleven genotypes including 5 donor and 6 recipient backgrounds was studied using genetic diversity and Principal Component Analysis (PCA). The UPGMA (unweighted pair group method with arithmetic mean) cluster analysis showed that the 11 rice genotypes were divided into two major groups (Fig. 3). All the recipients except the MTU1010 were present in Group I. The donors along with the upland adapted genotype MTU1010 constituted the Group II, which is further divided into two subgroups. The subgroup I had MTU1010, where, the subgroup II had other five donor backgrounds. The genotypes with *Aus* background Aus344, N22 and IRGC128442 were present in one subgroup whereas, the *indica* genotypes Kula Karuppan and NCS237 were present in another subgroup. Similarly, the recipient PR128 and PR129 were present in one subgroup and PR121, PR126 and Pusa Basmati 1509 in another subgroup.

### Phenotypic Validation of the KASP Assays

**Fig. 4:**
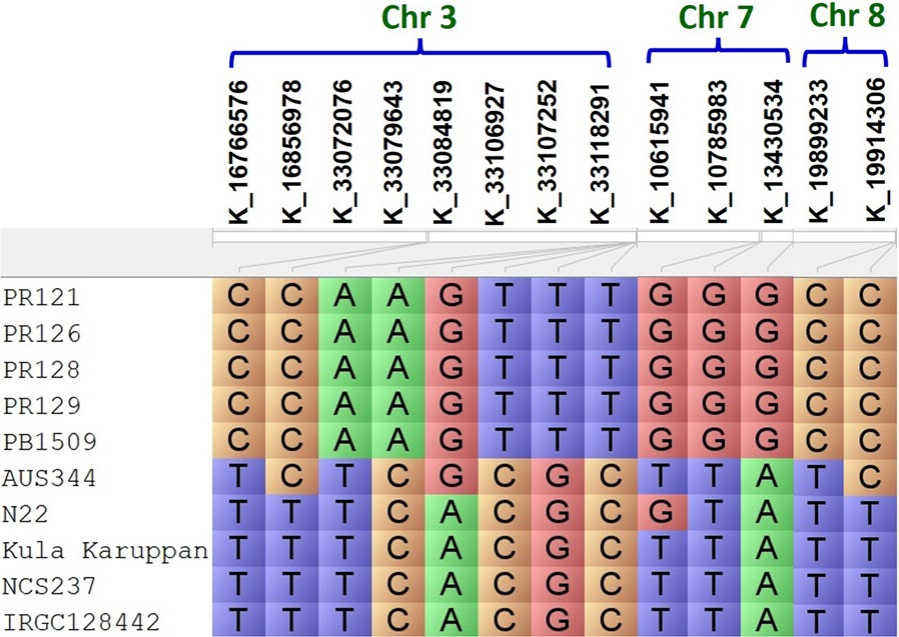
The allelic constitution of the 10 genotypes including 5 donors (Aus344, N22, Kula Karuppan, NCS237, IRGC 128442) and 5 recipient background (PR121, PR126, PR128, PR129, Pusa Basmati 1509) for the 13 KASP assays that showed polymorphism across all the recipient backgrounds associated with traits improving germination of rice under deep sown direct seeded cultivation conditions

All the 54 KASP assays which produced satisfactory results in parental polymorphism survey of the eleven genotypes were validated against the phenotypic performance of the F_3_:F_4_ and BC_3_F_2:3_ progenies. The allelic patterns of the ten genotypes for the 13 KASP assays that showed polymorphism across all the recipient backgrounds associated with traits improving germination of rice under deep sown direct seeded cultivation conditions is presented in Fig. 4. The allelic effects of the 54 KASP assays on the mean phenotypic values of the F_3_:F_4_ and BC_3_F_2:3_ progenies were assessed using the Kruskal–Wallis test and described in Table 3. The allelic effect for the KASP assays were found significant at *P* ≤ 0.05 in both the F_3_:F_4_ and BC_3_F_2:3_ progenies. The 54 phenotypically validated KASP assays include 16 assays for the genomic region associated with % germination and mesocotyl elongation on chromosome 3, 9 assays for the genomic region on chromosome 4, 23 assays for chromosome 7 and 6 assays for chromosome 8 (Table 3).

**Fig. 5:**
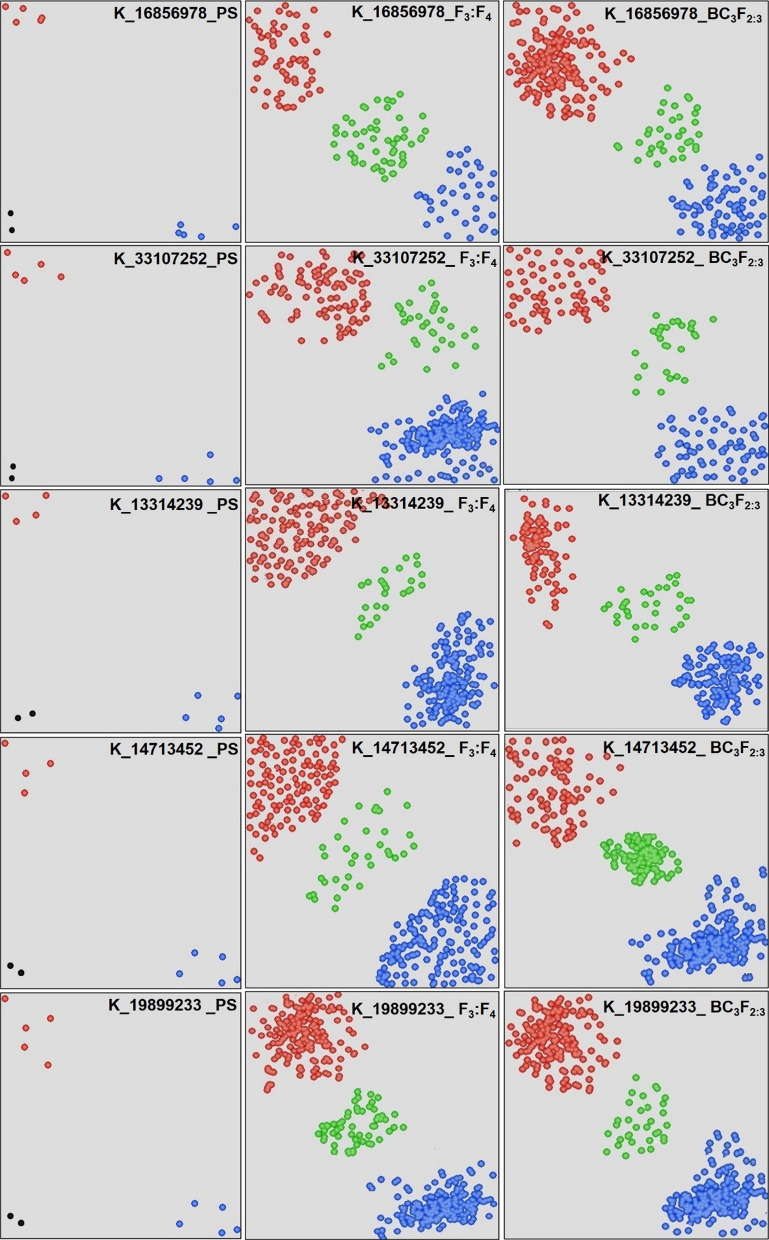
The pictorial representation of the KASP assays conducted on the including 5 donors (Aus344, N22, Kula Karuppan, NCS237, IRGC 128442) and 5 recipient background (PR121, PR126, PR128, PR129, Pusa Basmati 1509) used to develop the breeding panel and KASP assays on the breeding panel constituting F_3_:F_4_ and BC_3_F_2:3_ progenies. PS: polymorphism survey on the 10 genotypes. Blue color indicates the donor allele, red color indicates the recipient allele and green color indicates the heterozygotes

The few examples of KASP assays on the F_3_:F_4_ and BC_3_F_2:3_ progenies are presented in Fig. 5. The alleles associated with the improved germination and mesocotyl elongation showed significant improvement in germination and longer mesocotyl under deep sown direct seeded cultivation conditions. The F_3:4_ progenies carrying the alleles for improved germination showed 56.37–81.26% germination and 3.65–5.7 cm mesocotyl length compared to progenies carrying reference alleles 15.02–36.13% germination and 0.8–1.81 cm mesocotyl length when sown at 10 cm deep from the soil surface (Table 1). Similarly, the BC_3_F_2:3_ progenies carrying the alleles for improved germination showed 54.65–83.3% germination and 3.19–5.84 cm mesocotyl length compared to progenies carrying reference alleles 14.26–31.04% germination and 0.5–1.6 cm mesocotyl length when sown at 10 cm deep from the soil surface (Table 1).

### Reliability and Selection of KASP Assays

**Fig. 6:**
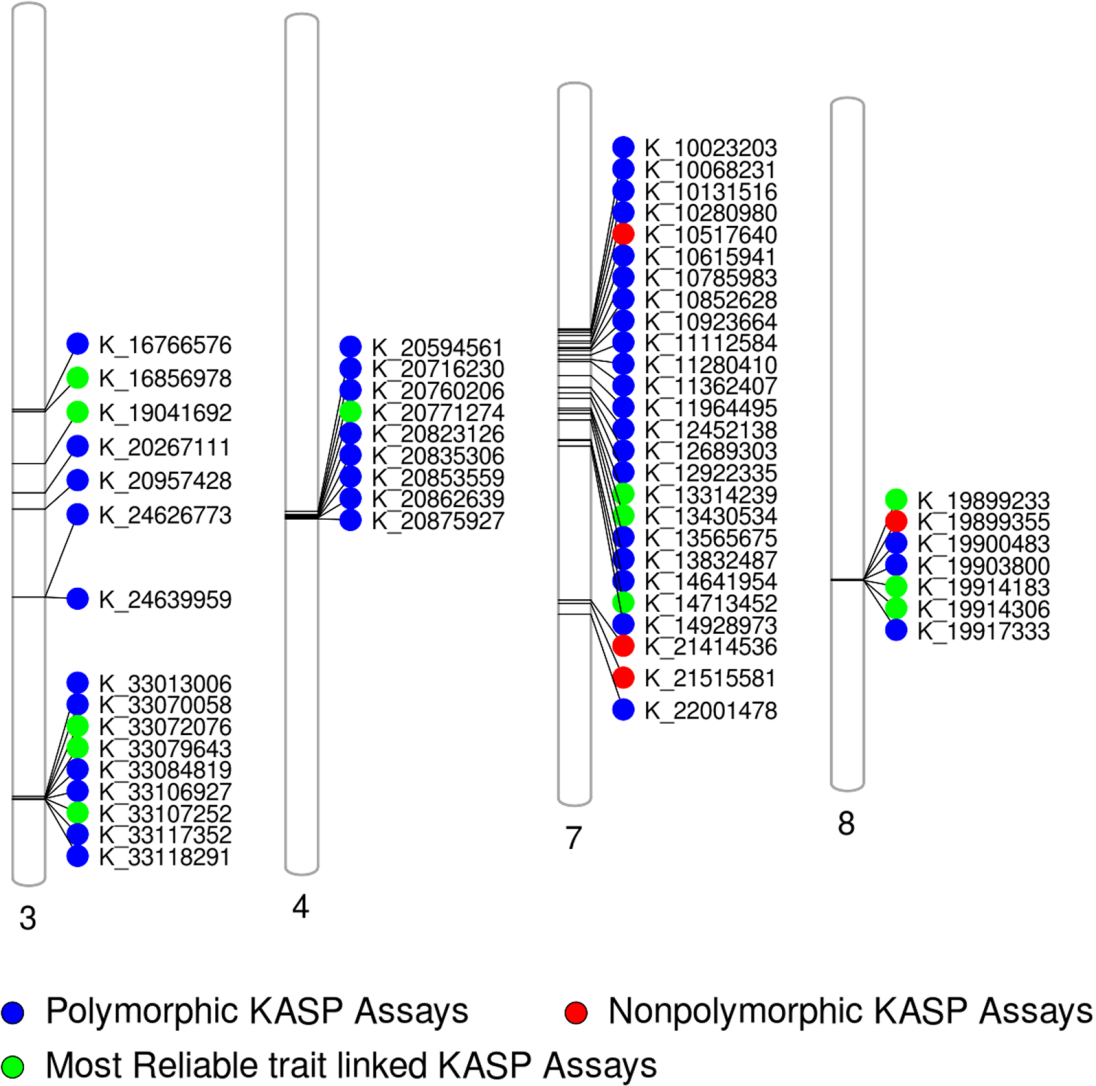
Schematic representation of the distribution of all the designed 58 KASP assays associated with seedling vigor traits along the four chromosomes of rice. The alternate SNP ID (K_followed by numeric value) showing genomic position in base pairs representing the physical position of the SNPs on the chromosome. The numbers below each chromosome indicate chromosome numbers. The four KASP assays with red color indicate KASP assays that were non polymorphic in parental survey. The remaining 54 KASP assays were polymorphic (blue color) and the green color indicates the most reliable 12 KASP (out of 54 polymorphic KASP assays) including 5 KASP on chromosome 3, 1 on chromosome 4, 3 on chromosome 7 and 3 on chromosome 8

The designed KASP assays were validated first on a set of 11 parents followed by the second level validation on the 15–20 predicted F_1_s plants developed from each cross considered in the present study. The third validation was carried out on F_3_:F_4_ and BC_3_F_2:3_ progenies. Further, the repeatability of the KASP assays was accessed on a set of random 50 samples from F_3_:F_4_ and 80–100 samples from BC_3_F_2:3_ progenies using 10 random markers. Based on the FPR, FNR, KASP utility in different genetic backgrounds and significant differences in the phenotypic values of the positive (desirable) and negative (undesirable) traits, a total of 12 KASP assays i.e. K_16856978, K_19041692, K_33072076, K_33079643, K_33107252, K_20771274, K_13314239, K_13430534, K_14713452, K_19899233, K_19914183 and K_19914306 have been selected. These 12 KASP include 5 KASP on chromosome 3, 1 on chromosome 4, 3 on chromosome 7 and 3 on chromosome 8. The R^2^ and p value calculated using single marker analysis in WinQTLCart V2.5 (Wang et al. 2012) of the 12 KASP assays are presented in Additional file 1: Table S4. The schematic representation of the distribution of KASP assays associated with seedling vigor traits on the chromosomes 3, 4, 7 and 8 of rice is presented in Fig. 6.

## Discussion

Development of molecular marker linked to the phenotypically important trait such as seedling vigor under deep sown direct seeded cultivation conditions are of great importance especially when trait phenotyping is laborious and difficult. In rice, where a strong focus on development of DSR adapted rice varieties has led to the introgression of various QTLs/gene providing adaptability to rice under DSR (Menard et al. 2021). Evaluation of below ground traits is not always straightforward because of various factors including soil, environment and technical/manual error and difficulties in measurement of traits such as mesocotyl elongation. Recent advancement in genomics offers several genomics-assisted breeding strategies such as the use of molecular markers to overcome these problems. Since 1980s, the breeders employed various kinds of molecular markers in cereal breeding, such as, RAPD (Random Amplified Polymorphic DNA), RFLP (Restriction Fragment Length Polymorphism), AFLP (Amplified Fragment Length Polymorphism), SSR (Simple Sequence Repeats) and STS (Sequence Tagged Sites) markers. The use of molecular markers has been successfully reported in crops rice (Jena and Mackill 2008), maize (Prasanna et al. 2010), wheat (Miedaner and Korzun 2012), barley (Miedaner and Korzun 2012), and sorghum (Mohamed et al. 2014; Rooney and Klein, 2000), for several traits to improve the efficiency of traditional breeding.

With the rapid progress in genomics, several initiatives including high throughput sequencing, identification of genomic regions associated with traits of interest, transcriptome or RNA sequencing have facilitated the development of functional molecular markers linked with the functional variants governing the trait variation. The identification of traits linked alleles/markers of choice are underway to achieve the targets in modern genomics-assisted breeding programs (Varshney et al. 2005). The concept of development of highly accurate SNP has now provided opportunities to target allelic variation improving yield and adaptability of rice under DSR. The whole genome resequencing of 11 genotypes in the present study provides about 2.8 million different types of SNP information which will help the breeders in mining the useful information about the SNPs. The high-throughput and cost-effective whole genome sequencing platform used in the present study to develop the trait linked KASP assays may help to maximize the genetic gains especially for complex traits under DSR (Semagn et al. 2014; Zhao et al. 2014). Therefore, the identification of the core trait-linked significant SNPs is must in genomics-assisted breeding. Till date, the diagnostic markers related to abiotic-biotic stress tolerance resistance such as *rtsv1*, *Xa4*, *xa5*, *xa13*, *Xa23*, *Xa21*, *Xa7,* Sub1A, (Lee et al. 2010*;* Li et al. 2001; Iyer and McCouch 2004; Dilla-Ermita et al. 2017; Chu et al. 2006; Peng et al 2015; Septiningsih et al. 2009) root traits improving nutrient uptake under DSR such as *qNR*_*4.1*_*, qNR*_*5.1*_*, qRHD*_*1.1*_*, qRHD*_*5.1*_*,* grain yield under DSR such as *qGY*_*1.1*_*, qGY*_*6.1*_*, qGY*_*10.1*_*,* grain yield under reproductive stage drought stress such as *qDTY*_*1.1*_*, qDTY*_*2.1*_*, qDTY*_*3.1*_*, qDTY*_*12.1*_ (Sandhu et al. 2022) and quality traits such as *ALK, Wx, GS3 Pikh*, *GW5*, and *CHALK5* (Gao et al. 2003; Bao et al. 2006; Dobo et al. 2010; Teng et al. 2017; Takano-Kai et al. 2009; Yang et al. 2019) have been reported.

The major challenge faced in designing the KASP assay was to identify the significant SNPs specifically linked with the particular donor/trait of interest and polymorphic with the multiple recipient backgrounds to be further used for the genomics-assisted breeding program. Finally, 54 KASP assays out of the 58 successfully-designed KASP were able to display the diversity at the loci. The 54 promising KASP assays with significant p-value being reported here showed significant association with the relevant phenotypes in the diverse donor/recipient backgrounds, recombinant and nearly isogenic breeding populations panel, thus revealing their potential application in the DSR breeding programs. The 54 polymorphic KASP assays fulfilled the criterion of quality control, allelic variations of the targeted donor to the recipient backgrounds, and strong association with the key/functional genes associated with traits improving seedling vigor under DSR. To the best of our knowledge the present study is the first report targeting development of trait-based KASP assays for the traits improving germination and mesocotyl length of rice under deep-sown DSR.

The genotyping results of the F_1_ plants, validation of KASP assays on multiple biparental populations and the better confidence values with very low false positive and false negative rates demonstrated the high levels of repeatability, accuracy, and the robustness of the KASP assays developed in the present study. These results are comparable to the results reported in other panels and genotyping platforms (Simen et al. 2015; Misyura et al. 2016; Thomson et al. 2017; Cai et al. 2017). The mean repeatability of KASP assays estimated in the present study was about 99%, the 1% dissimilarities between the predicted and the F_1_ calls could be explained by the genotypic errors of KASP assays. The accuracy and robustness of the KASP assays to call the heterozygous genotypes makes them suitable for genotyping the segregating populations, marker assisted backcross populations and to make genomic prediction in the segregating populations.

The selected 12 KASP assays with significant p-value and phenotypic variance (R^2^) (Additional file 1: Table S4) provide a platform for the foreground marker-assisted selection/introgression of traits improving germination of rice under deep sown DSR conditions. The selected 12 KASP array may be useful in constructing a set of nearly isogenic lines suitable for the deep sown DSR cultivation as the identified significant SNPs can be used to select the favourable alleles in a wide range of genetic backgrounds. The selected 12 tightly linked set of KASP assays can also be used for dissecting the linkage drag. The predictive abilities of the selected KASP assays obtained in this study suggest that these assays may be sufficient and cost-effective for the screening of germplasm possessing traits improving seedling vigor in deep sown DSR situation. The detection of haplotypes around the target favourable alleles can further be utilized for the fine genetic dissection of the genomic regions near the targeted genomic region.

We conducted an examination of how the SNP variants influence the protein structures to gain insights. Among the 54 SNPs selected for KASP assay design, we identified 25 located in intergenic regions, 13 within genic regions but situated in the introns of their respective genes, 4 within the untranslated regions (UTRs) of genes, and 7 within the coding regions of genes that produce translated proteins. Upon additional scrutiny, it was observed that 2 out of the 7 SNPs found in the exon regions led to synonymous mutations, while the remaining 5 resulted in missense variants. Further, we used SIFT (Sorting Intolerant From Tolerant) tool for predicting whether the missense variants are likely to affect the protein function based on sequence homology and the physico-chemical similarity between the alternate amino acids. Out of the 5 variants, 2 got a SIFT score of less than 0.05 thus indicating a possible deleterious effect of the SNP variants to the protein structure (Additional file 1: Table S3). In future, we are planning to do functional studies for the 2 genes (*LOC_Os04g34290* and *LOC_Os08g32100*) containing our 2 validated SNPs causing a possible deleterious mutation for the protein product.

**Fig. 7:**
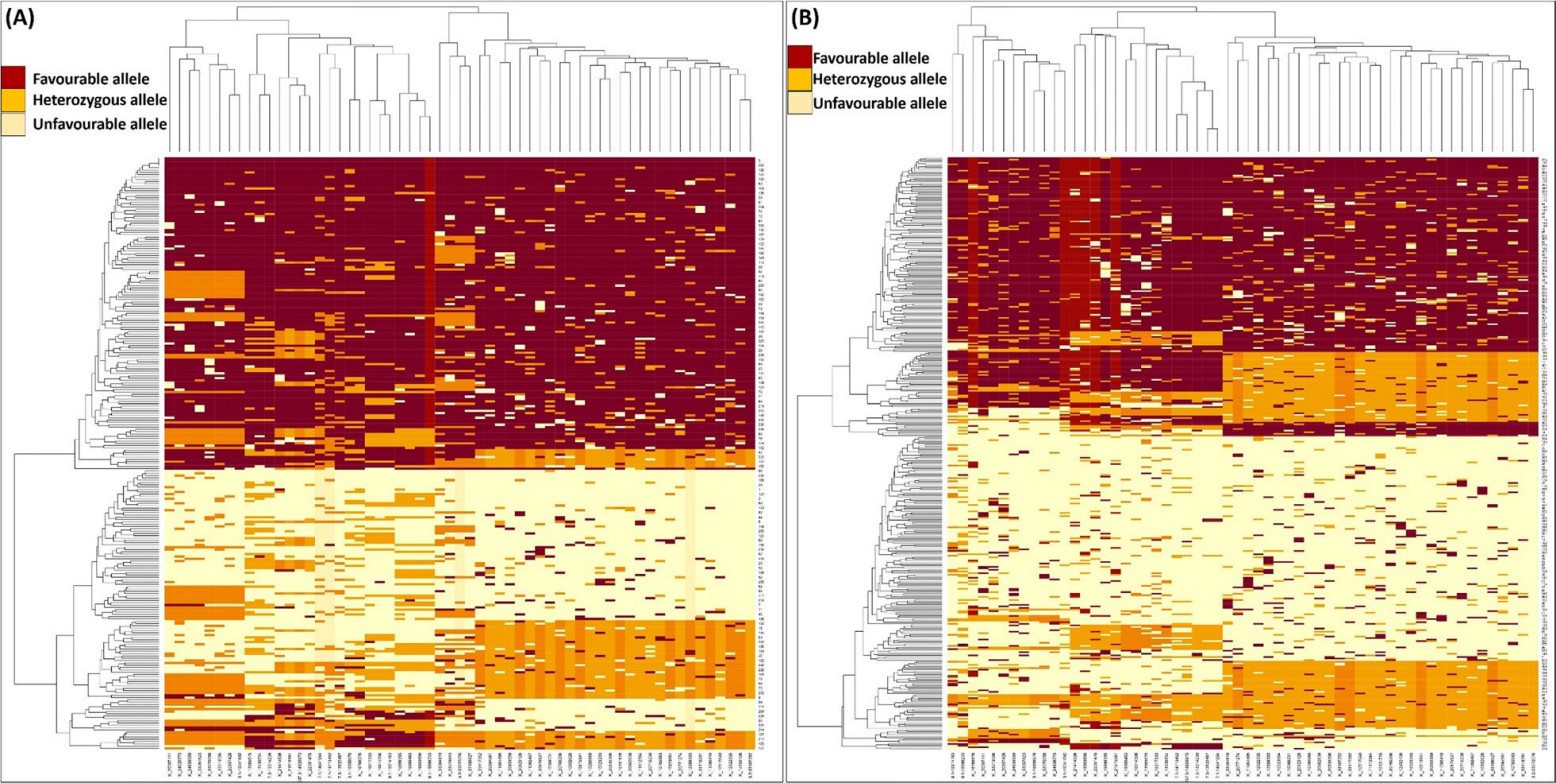
The heat map indicating the frequency of favourable alleles associated with traits linked with seedling vigor traits in deep sown direct-seeded rice **A** the F_3_:F_4_ progenies and **B** the BC_3_F_2:3_ progenies

The identified and validated KASP associated with seedling emergence would be desirable for marker-assisted introgression of traits providing adaptation to rice when sown deep under DSR into high-yielding modern cultivars. The heat map of the F_3_:F_4_ (Fig. 7A) and BC_3_F_2:3_ (Fig. 7B) indicating the frequency of the favorable alleles associated with germination of rice when sown deep. Development of genotypes with high seedling emergence under deep-sowing and tolerance to low oxygen during the seedling germination when the deeply sown rice seeds receive the unexpected early rains, as well as the high-vigor, weed competitiveness, and yield potential would likely be a successful strategy for DSR breeding. This will lead to the development of improved cultivars that are well-adapted to DSR and for making the long-term genetic gains.

### Supplementary Information

**Additional file 1: Table S1.** The detailed information on the number of plants from each cross used to validate the KASP assay.** Table S2.** Summary of the whole genome resequencing data. Chromosome wise distribution and mapping statistics of diverse rice accessions used in this study.** Table S3.** The detailed information on the genomic location of validated KASP markers within the MSUv7 gene models (http://rice.plantbiology.msu.edu).** Table S4.** The R^2^ and p value calculated using single marker analysis in WinQTLCart V2.5 (Wang et al. 2012) of the selected 12 KASP assays.

## Data Availability

The required data has been included in the supplementary information of the manuscript.
