# Supplementary material for: Development of Novel KASP Markers for Improved Germination in Deep-Sown Direct Seeded Rice
Source: Rice (N Y). 2024 May 10;17:33. doi: 10.1186/s12284-024-00711-1 (PMC11087395; doi:10.1186/s12284-024-00711-1)
Supplement: Supplementary file 1 — Additional file 1: Table S1. The detailed information on the number of plants from each cross used to validate the KASP assay. Table S2. Summary of the whole genome resequencing data. Chromosome wise distribution and mapping statistics of diverse rice accessions used in this study. Table S3. The detailed information on the genomic location of validated KASP markers within the MSUv7 gene models (http://rice.plantbiology.msu.edu). Table S4. The R2 and p value calculated using single marker analysis in WinQTLCart V2.5 (Wang et al. 2012) of the selected 12 KASP assays. [file 12284_2024_711_MOESM1_ESM.docx]

**Additional file 1: Table S1. The detailed information on the number of plants from each cross used to validate the KASP assay.**

| RILs | Cross name | Number of plants |
| --- | --- | --- |
|  | PR121/N22 | 22 |
|  | PR126/N22 | 22 |
|  | PR128/N22 | 47 |
|  | PB1509/N22 | 47 |
|  | PR121/Aus344 | 27 |
|  | PR129/Aus344 | 42 |
|  | PR128/IRGC128442 | 49 |
|  |  |  |
| NILs |  |  |
|  |  |  |
|  | N22/4*PR121 | 55 |
|  | N22/4*PR126 | 32 |
|  | N22/4*PR128 | 46 |
|  | N22/4*PB1509 | 55 |
|  | Aus344/4*PR121 | 55 |
|  | Aus344/4*PR129 | 45 |
|  | IRGC128442/4*PR126 | 370 |
|  | IRGC128442/4*PR128 | 55 |

**Additional file 1: Table S2. Summary of the whole genome resequencing data. Chromosome wise distribution and mapping statistics of diverse rice accessions used in this study.**

| **Designation** | **No. of Reads** | **Sequence length** | **%GC** | **Total bp** | **Mapping** | **Variants at 10x** |
| --- | --- | --- | --- | --- | --- | --- |
|  |  |  |  |  | **percent** |  |
| PR 126 | 48752804 | 150 | 44 | 14625841200 | 98.4 | 3324005 |
| PR 121 | 36194134 | 150 | 43 | 10858240200 | 98.26 | 3275238 |
| PR 128 | 51385861 | 150 | 44 | 15415758300 | 98.37 | 3305965 |
| PR 129 | 39340451 | 150 | 43 | 11802135300 | 98.36 | 3220427 |
| MTU 1010 | 44538343 | 150 | 43 | 13361502900 | 98.27 | 3345195 |
| Pusa Basmati 1509 | 44696388 | 150 | 44 | 13408916400 | 97.71 | 3401748 |
| AUS344 | 43373919 | 161 | 48 | 13966401918 | 98.24 | 2644557 |
| N22 | 24902576 | 161 | 44 | 8018629472 | 98.41 | 3246340 |
| Kula Karuppan | 25562970 | 161 | 45 | 8231276340 | 98.46 | 184335 |
| NCS237 | 27672112 | 161 | 43 | 8910420064 | 97.5 | 248356 |
| IRGC128442 | 23662221 | 161 | 45 | 7619235162 | 98.45 | 2742815 |
| Total | **410081779** |  |  | **126218357256** |  | **28938981** |
| Average per sample | **37280162** |  |  | **11474396114** | **98.2209091** | **2630816** |

**Additional file 1: Table S3: The detailed information on the genomic location of validated KASP markers within the MSUv7 gene models (**[**http://rice.plantbiology.msu.edu**](http://rice.plantbiology.msu.edu)**)**

| **Original SNP ID** | **Marker ID** | **Chr** | **SNP position IRGSP1.0** | **Allele** | **Intergenic or Genic (LOC) marker** | **Gene product name** | **Coding/non coding region** | **Change in amino acid** | | **Codons** | **SIFT** |
| --- | --- | --- | --- | --- | --- | --- | --- | --- | --- | --- | --- |
| chr03:16766576 | K_16766576 | 3 | 16766576 | C/T | *LOC_Os03g29420* | hypothetical protein | Intergenic Variant |  |  |  |  |
| chr03:16856978 | K_16856978 | 3 | 16856978 | C/T | *LOC_Os03g29570* | mps one binder kinase activator-like 1A, putative, expressed | Intron variant |  |  |  |  |
| chr03:19041692 | K_19041692 | 3 | 19041692 | C/T | *LOC_Os03g33290* | retrotransposon protein, putative, unclassified, expressed | Intergenic Variant |  |  |  |  |
| chr03:20267111 | K_20267111 | 3 | 20267111 | T/C | *LOC_Os03g36560* | peroxidase precursor, putative, expressed | Intron variant |  |  |  |  |
| chr03:20957428 | K_20957428 | 3 | 20957428 | A/T | *LOC_Os03g37790* | hAT dimerisation domain-containing protein, putative, expressed | Intergenic Variant |  |  |  |  |
| chr03:24626773 | K_24626773 | 3 | 24626773 | T/G | *LOC_Os03g43890* | WD domain, G-beta repeat domain containing protein, expressed | Intron variant |  |  |  |  |
| chr03:24639959 | K_24639959 | 3 | 24639959 | C/T | *LOC_Os03g43910* | expressed protein | Intron variant |  |  |  |  |
| chr03:33013006 | K_33013006 | 3 | 33013006 | A/C | *LOC_Os03g57950* | type I inositol-1,4,5-trisphosphate 5-phosphatase, putative, expressed | Coding missense variant | N/H | Asparagine/  Histidine | **A**AC/  **C**AC | 0.1 |
| chr03:33070058 | K_33070058 | 3 | 33070058 | A/C | *LOC_Os03g58080* | BT1 family protein, putative, expressed | Intron variant |  |  |  |  |
| chr03:33072076 | K_33072076 | 3 | 33072076 | A/T | *LOC_Os03g58090* | expressed protein | 3-prime UTR variant |  |  |  |  |
| chr03:33079643 | K_33079643 | 3 | 33079643 | A/C | *LOC_Os03g58100* | pentatricopeptide, putative, expressed | Coding synonymous variant |  |  |  |  |
| chr03:33084819 | K_33084819 | 3 | 33084819 | G/A | *LOC_Os03g58110* | uncharacterized protein At4g06744 precursor, putative, expressed | Intron variant |  |  |  |  |
| chr03:33106927 | K_33106927 | 3 | 33106927 | T/C | *LOC_Os03g58160* | heat stress transcription factor, putative, expressed | Intron variant |  |  |  |  |
| chr03:33107252 | K_33107252 | 3 | 33107252 | T/G | *LOC_Os03g58160* | heat stress transcription factor, putative, expressed | Intron variant |  |  |  |  |
| chr03:33117352 | K_33117352 | 3 | 33117352 | C/T | *LOC_Os03g58170* | stem-specific protein TSJT1, putative, expressed | Intron variant |  |  |  |  |
| chr03:33118291 | K_33118291 | 3 | 33118291 | T/C | *LOC_Os03g58170* | stem-specific protein TSJT1, putative, expressed | Downstream gene variant |  |  |  |  |
| chr04:20594561 | K_20594561 | 4 | 20594561 | A/G | *LOC_Os04g34010* | aluminum-activated malate transporter, putative, expressed | Intron variant |  |  |  |  |
| chr04:20716230 | K_20716230 | 4 | 20716230 | T/C | *LOC_Os04g34180* | expressed protein | Intergenic variant |  |  |  |  |
| chr04:20760206 | K_20760206 | 4 | 20760206 | G/A | *LOC_Os04g34270* | serine/threonine-protein kinase receptor precursor, putative, expressed | Coding missense variant | D/N | Aspartic acid/Asparagine | **G**AC/**A**AC | 0.06 |
| chr04:20771274 | K_20771274 | 4 | 20771274 | G/A | *LOC_Os04g34290* | protein kinase, putative, expressed | Coding missense variant | D/N | Aspartic acid/Asparagine | **G**AT/**A**AT | 0.03 |
| chr04:20823126 | K_20823126 | 4 | 20823126 | G/A | *LOC_Os04g34380* | expressed protein | Intergenic variant |  |  |  |  |
| chr04:20835306 | K_20835306 | 4 | 20835306 | T/A | *LOC_Os04g34410* | serine/threonine-protein kinase receptor precursor, putative, expressed | Intergenic variant |  |  |  |  |
| chr04:20853559 | K_20853559 | 4 | 20853559 | A/T | *LOC_Os04g34440* | ubiquitin interaction motif-containing protein, putative, expressed | Splice region variant |  |  |  |  |
| chr04:20862639 | K_20862639 | 4 | 20862639 | C/A | *LOC_Os04g34450* | expressed protein | 5’ UTR variant |  |  |  |  |
| chr04:20875927 | K_20875927 | 4 | 20875927 | A/G | *LOC_Os04g34460* | PAP fibrillin family domain containing protein, expressed | 3’ UTR Variant |  |  |  |  |
| chr07:10023203 | K_10023203 | 7 | 10023203 | G/T | intergenic |  | Intergenic variant |  |  |  |  |
| chr07:10068231 | K_10068231 | 7 | 10068231 | T/A | intergenic |  | Intergenic variant |  |  |  |  |
| chr07:10131516 | K_10131516 | 7 | 10131516 | T/C | LOC_Os07g17210 | FAS1, putative, expressed | Synonymous variant |  |  |  |  |
| chr07:10280980 | K_10280980 | 7 | 10280980 | T/A | intergenic |  | Intergenic variant |  |  |  |  |
| chr07:10615941 | K_10615941 | 7 | 10615941 | G/T | intergenic |  | Intergenic variant |  |  |  |  |
| chr07:10785983 | K_10785983 | 7 | 10785983 | G/T | *LOC_Os07g18190* | retrotransposon protein, putative, Ty3-gypsy subclass, expressed | Intergenic variant |  |  |  |  |
| chr07:10852628 | K_10852628 | 7 | 10852628 | A/T | intergenic |  | Intergenic variant |  |  |  |  |
| chr07:10923664 | K_10923664 | 7 | 10923664 | C/T | *LOC_Os07g18460* | expressed protein | Intergenic variant |  |  |  |  |
| chr07:11112584 | K_11112584 | 7 | 11112584 | C/T | *LOC_Os07g18780* | retrotransposon protein, putative, Ty1-copia subclass, expressed | Intergenic variant |  |  |  |  |
| chr07:11280410 | K_11280410 | 7 | 11280410 | C/A | *LOC_Os07g19060* | transport protein particle component, Bet3, domain containing protein, expressed | 3’ UTR Variant |  |  |  |  |
| chr07:11362407 | K_11362407 | 7 | 11362407 | C/T | *LOC_Os07g19190* | ribosomal protein L24, putative, expressed | Intergenic variant |  |  |  |  |
| chr07:11964495 | K_11964495 | 7 | 11964495 | C/A | intergenic |  | Intergenic variant |  |  |  |  |
| chr07:12452138 | K_12452138 | 7 | 12452138 | G/T | intergenic |  | Intergenic variant |  |  |  |  |
| chr07:12689303 | K_12689303 | 7 | 12689303 | C/T | *LOC_Os07g22550* | retrotransposon protein, putative, Ty3-gypsy subclass, expressed | Intergenic variant |  |  |  |  |
| chr07:12922335 | K_12922335 | 7 | 12922335 | A/G | *LOC_Os07g22930* | starch synthase, putative, expressed | Intron variant |  |  |  |  |
| chr07:13314239 | K_13314239 | 7 | 13314239 | T/C | *LOC_Os07g23550* | expressed protein | Intron variant |  |  |  |  |
| chr07:13430534 | K_13430534 | 7 | 13430534 | G/A | intergenic |  | Intergenic variant |  |  |  |  |
| chr07:13565675 | K_13565675 | 7 | 13565675 | C/G | intergenic |  | Intergenic variant |  |  |  |  |
| chr07:13832487 | K_13832487 | 7 | 13832487 | A/G | *LOC_Os07g24320* | retrotransposon protein, putative, unclassified, expressed | Intergenic variant |  |  |  |  |
| chr07:14641954 | K_14641954 | 7 | 14641954 | G/A | *LOC_Os07g25550* | hAT dimerisation domain-containing protein, putative, expressed | Intergenic variant |  |  |  |  |
| chr07:14713452 | K_14713452 | 7 | 14713452 | G/A | *LOC_Os07g25660* | expressed protein | Intergenic variant |  |  |  |  |
| chr07:14928973 | K_14928973 | 7 | 14928973 | C/T | *LOC_Os07g26020* | retrotransposon protein, putative, unclassified, expressed | Intergenic variant |  |  |  |  |
| chr07:22001478 | K_22001478 | 7 | 22001478 | G/A | *LOC_Os07g36720* | retrotransposon protein, putative, unclassified, expressed | Intergenic variant |  |  |  |  |
| chr08:19899233 | K_19899233 | 8 | 19899233 | C/T | *LOC_Os08g32090* | DEAD-box ATP-dependent RNA helicase, putative, expressed | Intron variant |  |  |  |  |
| chr08:19900483 | K_19900483 | 8 | 19900483 | T/A | *LOC_Os08g32090* | DEAD-box ATP-dependent RNA helicase, putative, expressed | Intron variant |  |  |  |  |
| chr08:19903800 | K_19903800 | 8 | 19903800 | C/A | *LOC_Os08g32090* | DEAD-box ATP-dependent RNA helicase, putative, expressed | Intron variant |  |  |  |  |
| chr08:19914183 | K_19914183 | 8 | 19914183 | T/C | *LOC_Os08g32100* | transposon protein, putative, CACTA, En/Spm sub-class, expressed | Coding missense variant | N/S | Asparagine/  Serine | A**A**C/A**G**C | 0.03 |
| chr08:19914306 | K_19914306 | 8 | 19914306 | C/T | *LOC_Os08g32100* | transposon protein, putative, CACTA, En/Spm sub-class, expressed | Coding missense variant | S/N | Serine/  Asparagine | A**G**T/A**A**T | 0.56 |
| chr08:19917333 | K_19917333 | 8 | 19917333 | G/A | *LOC_Os08g32100* | transposon protein, putative, CACTA, En/Spm sub-class, expressed | Intron variant |  |  |  |  |

**Additional file 1: Table S4: The R^2^ and p value calculated using single marker analysis in WinQTLCart V2.5 (Wang et al. 2012) of the selected 12 KASP assays**

| **Marker** | **Chr** | **Position** | **R^2^** | **p-value** |
| --- | --- | --- | --- | --- |
| K_16856978 | 3 | 16856978 | 0.6248 | **** |
| K_19041692 | 3 | 19041692 | 0.6937 | **** |
| K_33072076 | 3 | 33072076 | 0.61 | **** |
| K_33079643 | 3 | 33079643 | 0.6787 | **** |
| K_33107252 | 3 | 33107252 | 0.6655 | **** |
| K_20771274 | 4 | 20771274 | 0.6567 | **** |
| K_13314239 | 7 | 13314239 | 0.7145 | **** |
| K_13430534 | 7 | 13430534 | 0.7525 | **** |
| K_14713452 | 7 | 14713452 | 0.731 | **** |
| K_19899233 | 8 | 19899233 | 0.6614 | **** |
| K_19900483 | 8 | 19900483 | 0.6412 | **** |
| K_19914306 | 8 | 19914306 | 0.6443 | **** |

****significance at < 0.01% level
